# Deletion of *flgL* in *Mesorhizobium ciceri* USDA 3378 weakened competitive nodulation ability by reducing flagellum formation, biofilm formation, and extracellular polysaccharide secretion

**DOI:** 10.1128/aem.00931-26

**Published:** 2026-07-10

**Authors:** Keyu Chen, Cancan Zhu, Ke Li, Haoran Hao, Kaiwen Zhang, Youguo Li, Mitchell Andrews, Junjie Zhang

**Affiliations:** 1 College of Food and Bioengineering, Zhengzhou University of Light Industry 117776, Zhengzhou, Henan, People's Republic of China; 2 College of Life Science and Technology, Huazhong Agricultural University 47895, Wuhan, Hubei, China; 3 Faculty of Agriculture and Life Sciences, Lincoln University 2244, Lincoln, New Zealand; 4 Collaborative Innovation Center for Food Production and Safety of Henan Province, Zhengzhou, Henan, People's Republic of China; 5 Sanquan Food Co., Ltd., Zhengzhou, Henan, People's Republic of China; Indiana University Bloomington, Bloomington, Indiana, USA

**Keywords:** *Mesorhizobium ciceri*, *Mesorhizobium muleiense*, transcriptome, *flgL *gene, competitive nodulation

## Abstract

*Mesorhizobium ciceri* USDA 3378 has a competitive advantage over the indigenous *Mesorhizobium muleiense* CCBAU 83963 in nodulating chickpea (*Cicer arietinum* L.) in newly introduced planting areas in China. The underlying mechanisms for this dominance remain unclear. A comparison of the genomes of USDA 3378 and CCBAU 83963 revealed significantly more genes involved in flagellum production and cell movement in USDA 3378. USDA 3378 produced flagella, but CCBAU 83963 did not and showed lower motility, biofilm production, and extracellular polysaccharide secretion than USDA 3378. Transcriptome analysis of USDA 3378 under simulated symbiotic versus non-symbiotic conditions showed strong induction of nodulation genes and a broader transcriptional response among genes assigned to quorum sensing, chemotaxis, and flagellar assembly, with *flgL* (encoding a flagellar hook-associated family protein) being the only upregulated flagellar structural gene detected. A *flgL* mutant strain based on USDA 3378 (Δ*flgL*-3378) showed similar growth to USDA 3378 but was unable to produce flagella and exhibited concomitant reductions in motility, biofilm production, and extracellular polysaccharide secretion. Nodule occupancy by USDA 3378 was 100% when co-inoculated with CCBAU 83963. In contrast, nodule occupancy by Δ*flgL*-3378 was significantly reduced to 39.88% when co-inoculated with the wild-type USDA 3378. However, when co-inoculated with the indigenous strain CCBAU 83963, Δ*flgL*-3378 still showed a dominant occupancy of 82.8%. Transcriptome analysis of Δ*flgL*-3378 under the same comparison showed continued induction of nodulation genes and several flagellar system genes, an altered quorum-sensing-associated response, and no detectable chemotaxis-related differentially expressed genes. We conclude that *flgL* and flagella act as important contributors to the superior competitive nodulation ability of *M. ciceri* USDA 3378 over *M. muleiense* in chickpea, although other intrinsic genomic advantages likely contribute to its basal competitiveness

**IMPORTANCE:**

Chickpea is an important legume crop that depends on symbiotic rhizobia for biological nitrogen fixation. In newly introduced chickpea-growing regions of China, *Mesorhizobium ciceri* USDA 3378 shows a strong competitive advantage in nodulating chickpea compared with the indigenous strain *Mesorhizobium muleiense* CCBAU 83963, but the mechanisms underlying this advantage remain unclear. This study identifies the flagellar hook-associated gene *flgL* as an important contributor to the competitive nodulation ability of USDA 3378. Deletion of *flgL* abolished flagellum formation and reduced motility, biofilm formation, extracellular polysaccharide production, and competitive nodulation ability. However, the Δ*flgL* mutant still retained higher competitiveness than CCBAU 83963, indicating that additional motility-independent traits also contribute to the basal competitiveness of USDA 3378. These findings improve our understanding of the bacterial traits that influence rhizobial competitiveness and may help guide the development of more effective chickpea inoculants for diverse agricultural environments.

## INTRODUCTION

Chickpea (*Cicer arietinum* L.) is grown commercially as an annual grain legume crop in more than 50 countries across Asia, North Africa, Southern Europe, North America, and Australia (1). It ranks third behind soybean (*Glycine max*) and peanut (*Arachis hypogaea*) in global production among grain/oilseed legume crops (1, 2). The average annual planting area of chickpea in China is approximately 7,000 hectares, primarily in Xinjiang, Gansu, and Ningxia provinces. However, chickpea has been introduced into several other provinces in recent years with a view to increasing its production (3).

As for other grain legume crops, chickpea fixes atmospheric nitrogen via symbiotic bacteria (rhizobia) in root nodules, and this contributes to its production of high-protein seeds and overall yield (3). Chickpea shows specificity for rhizobial symbionts and is nodulated by only a few *Mesorhizobium* species. *Mesorhizobium ciceri* and *Mesorhizobium mediterraneum* are the dominant populations in western and South Asian countries, and *Mesorhizobium muleiense* and *Mesorhizobium wenxiniae* are the dominant populations in Northwest China (4–7). Strains of these four species show high similarity in sequences of their symbiosis genes *nodA*, *nodC*, and *nifH* (7, 8). Indeed, *M. muleiense* and *M. wenxiniae* are indigenous to China and likely obtained their *nod* and *nif* genes via horizontal transfer from introduced rhizobial species such as *M. ciceri* (8).

Previously, we compared *M. ciceri* USDA 3378 and *M. muleiense* CCBAU 83963 under different conditions to identify a competitive and efficient rhizobium inoculant strain for chickpea in the new production areas in China (9). *M. ciceri* USDA 3378 displayed nodule occupancy rates exceeding 80% in soils containing both strains. Also, in mixed applications in pots, *M. ciceri* USDA 3378 showed stronger attachment to chickpea roots. The objective of the current study was to gain greater understanding of how the introduced *M. ciceri* USDA 3378 has a competitive advantage over the indigenous *M. muleiense* CCBAU 83963 in nodulation of chickpea. Firstly, we compared the genomes of the two strains focusing on gene clusters related to six factors previously reported to be important in the nodulation process: flagellum production, chemotaxis, quorum sensing, type III secretion systems, extracellular polysaccharide production, and nodulation gene systems (10–13). The major difference between the two strains in this comparison was that there were more genes involved in flagellum production and cell movement in USDA 3378 than in CCBAU 83963. Further work tested the possibility that this difference in flagellar system genes could be the reason for the greater competitive nodulation ability of USDA 3378 compared to CCBAU 83963 for chickpea.

## RESULTS

### Genome comparison indicates differences in flagellar gene clusters between USDA 3378 and CCBAU 83963

**Fig 1:**
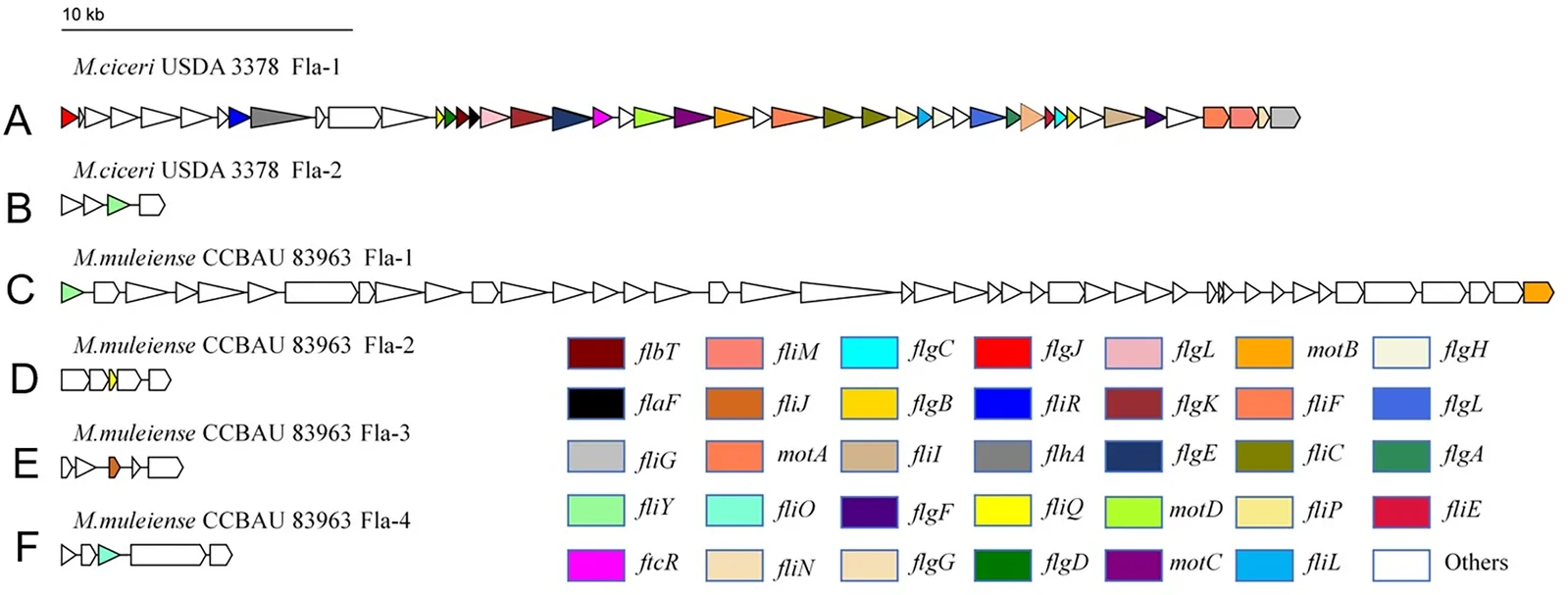
Map of the flagellar gene clusters of rhizobial strains *M. ciceri* USDA 3378 and *M. muleiense* CCBAU 83963. The color in each rectangle represents a gene corresponding to those in the gene cluster. The USDA 3378 genome contained 32 flagellum-related genes in two gene clusters (**A and B**), and the CCBAU 83963 genome contained five flagellum-related genes in four gene clusters (**C–F**).

A comparison of functional gene clusters related to the nodulation process in *M. ciceri* USDA 3378 and *M. muleiense* CCBAU 83963 showed little difference between the two strains for genes related to chemotaxis (Table S1), quorum sensing (Fig. S1A), extracellular polysaccharide production (Fig. S1B), type III secretion (Fig. S1C), and nodulation (Fig. S1D). However, there were substantially more genes involved in flagellum production and cell movement in USDA 3378 than in CCBAU 83963. The distribution of flagellum-related genes encoded in the genome was predicted in order to visualize the flagellum-related gene clusters in each strain (Fig. 1). The USDA 3378 genome contained 32 flagellum-related genes, comprising two clusters. One of these, the fla-1 gene cluster, was relatively conserved. The genome of CCBAU 83963 contained five flagellum-related genes scattered across four different gene clusters in different genomic regions. It was speculated that this difference in flagellar system genes could be the reason for the greater competitive nodulation ability of USDA 3378 compared to CCBAU 83963 in chickpea, and subsequent work tested this possibility.

### The *flgL* gene of USDA 3378 may be important to its competitive nodulation ability

**Fig 2:**
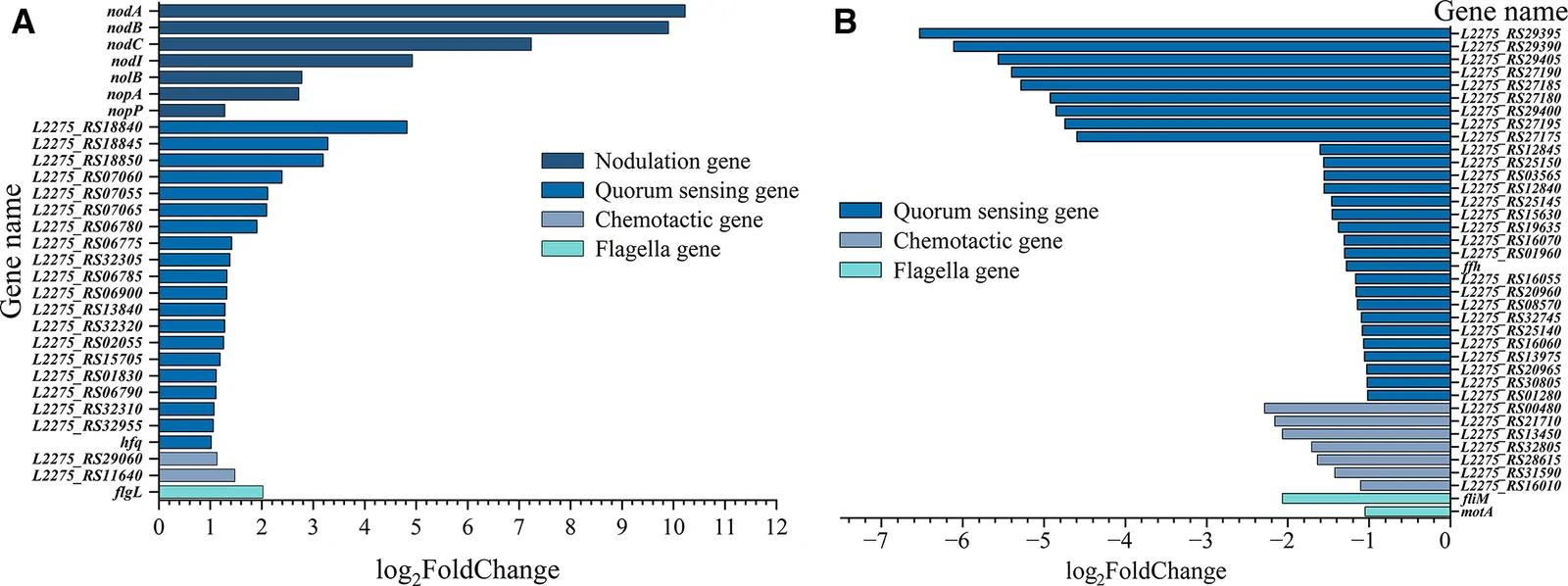
Differentially expressed genes (DEGs) related to nodulation, quorum sensing, chemotaxis, and flagellar functions in *M. ciceri* USDA 3378 under symbiotic environment-mimicking conditions. (**A**) Upregulated DEGs and (**B**) downregulated DEGs. Genes were classified as differentially expressed when meeting the thresholds of *q*-value < 0.05 and |fold change| > 1.

Transcriptome sequencing of USDA 3378 was performed under conditions designed to mimic the symbiotic environment. Upon the addition of chickpea seedlings, USDA 3378 showed strong induction of nodulation genes together with differential expression of genes assigned to quorum sensing, chemotaxis, and flagellar functions (Fig. 2). The upregulated expression of nodulation genes *nodA*, *nodB*, and *nodC*, which respond to “signals” (often specific flavonoids) from compatible legumes and encode the enzymes required for the synthesis of the core Nod factor structure—the signal molecule from the rhizobium that induces nodule morphogenesis in the legume—provided strong evidence that the experimental conditions mimicked symbiotic conditions. Among genes related to flagellum production, *flgL*, encoding a flagellar hook-associated family protein, was the only upregulated gene detected.

### Deletion of *flgL* does not affect the growth of *M. ciceri*

**Fig 3:**
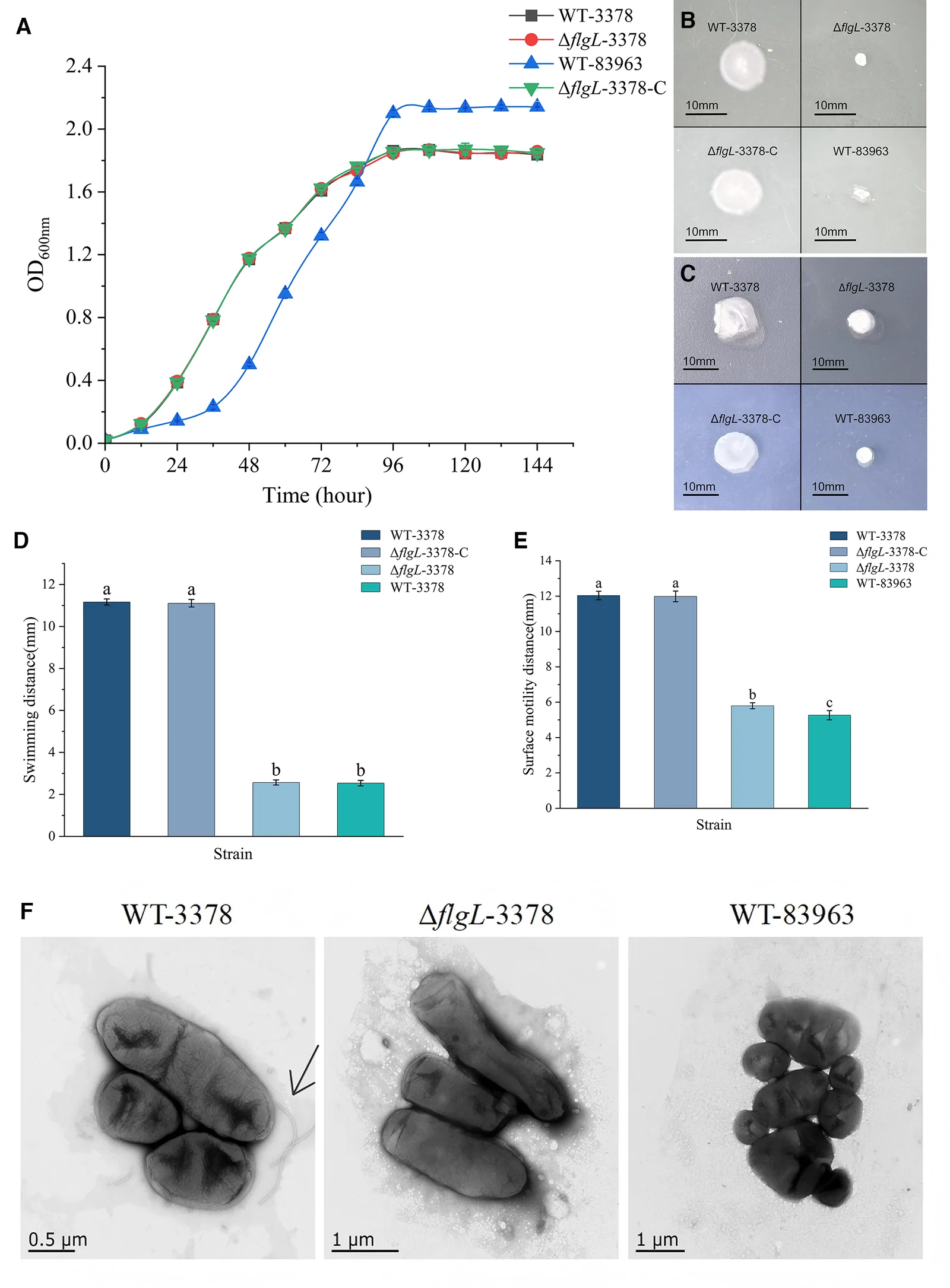
Growth curves (**A**), motility, and flagellum production (**B and C**) of rhizobial strains *M. ciceri* USDA 3378 (WT-3378), *M. ciceri* 3378 deficient in *flgL* (Δ*flgL*-3378), Δ*flgL*-3378 complementation strain (Δ*flgL*-3378-C), and *M. muleiense* CCBAU 83963 (WT-83963). (**D and E**) Data are presented as means (*n* = 3) ± standard deviations. Different letters indicate significant differences between treatments (*P* < 0.05) as determined by one-way analysis of variance. WT-3378 produced flagella (black arrow indicates flagellar structure) but Δ*flgL*-3378 and WT-83963 did not (**F**).

Homologous recombination was successfully used to delete *flgL* from USDA 3378. The upstream and downstream homologous arms of the *flgL* gene were amplified from the USDA 3378 genome, with lengths of 516 bp and 503 bp, respectively (Fig. S2A). These arms were linked to plasmid pCM351 by seamless cloning to construct the recombinant plasmid pCM351::*flgL*up-down. Colony PCR amplification of the 516 bp (Fig. S2B) and 503 bp (Fig. S2C) fragments, along with sequencing of the whole plasmid, confirmed the successful construction of pCM351::*flgL*up-down. Following biparental mating between *E. coli* S17-1 carrying the recombinant plasmid and wild-type USDA 3378 (WT-3378), mutant *M. ciceri* strains were identified by colony PCR. The wild-type strain (“control”) produced a band of 2,426 bp, while the mutant strain produced a band of 2,298 bp, consistent with the deletion of *flgL* (Fig. S2D). Sequences of the mutants were consistent with those expected, and we produced a *flgL* complementation strain of Δ*flgL*-3378, Δ*flgL*-3378-C (Fig. S3A through C). Growth of Δ*flgL*-3378 and WT-3378 at 28°C was similar for 144 h, indicating that the deletion of *flgL* did not affect the growth of *M. ciceri* (Fig. 3A).

### Deletion of *flgL* impaired flagellum formation and motility under both standard and host-stimulated conditions

Flagellum-based motility can improve the ability of rhizobia to establish symbiosis with the legume host and was tested for the Δ*flgL* strain. After 5 days of culture, the swimming motility distance of WT-3378 was 11.16 ± 0.15 mm (Fig. 3B and D). In contrast, Δ*flgL*-3378 and WT-83963 showed negligible expansion (2.57 ± 0.12 mm and 2.54 ± 0.13 mm, respectively), consistent with non-motile colony growth rather than active swimming (Fig. 3B and D). Similarly, the surface motility distances of WT-3378, Δ*flgL*-3378, and WT-83963 on 0.4% agarose MM medium plates were 12.03 ± 0.24 mm, 5.80 ± 0.11 mm, and 5.27 ± 0.26 mm, respectively (Fig. 3C and E). Values for both swimming and surface motility were similar for WT-3378 and Δ*flgL*-3378-C (Fig. 3B through D). These results indicate that motor ability was greater for WT-3378 than WT-83963, and deletion of the *flgL* gene substantially reduced the motor ability of *M. ciceri*.

The effect of the *flgL* gene mutation on the flagella of WT-3378 and whether strain WT-83963 had flagella were tested by transmission electron microscopy. WT-3378 showed slender flagellar structures, while Δ*flgL*-3378 and WT-83963 lacked flagella entirely (Fig. 3F).

To determine if host signals affect motility, we examined the swimming and surface motility of the strains on plates supplemented with 4% (vol/vol) unconcentrated chickpea root exudates. The results showed that the presence of root exudates significantly enhanced the motility of WT-3378 compared to the control (without exudates), resulting in larger swimming and swarming halos (Fig. S4). In contrast, the motility of the Δ*flgL*-3378 mutant remained unchanged in the presence of exudates. This indicates that host root exudates specifically stimulate the flagellum-dependent motility of *M. ciceri*.

### Biofilm formation and extracellular polysaccharide production are reduced in the Δ*flgL*-3378 mutant

**Fig 4:**
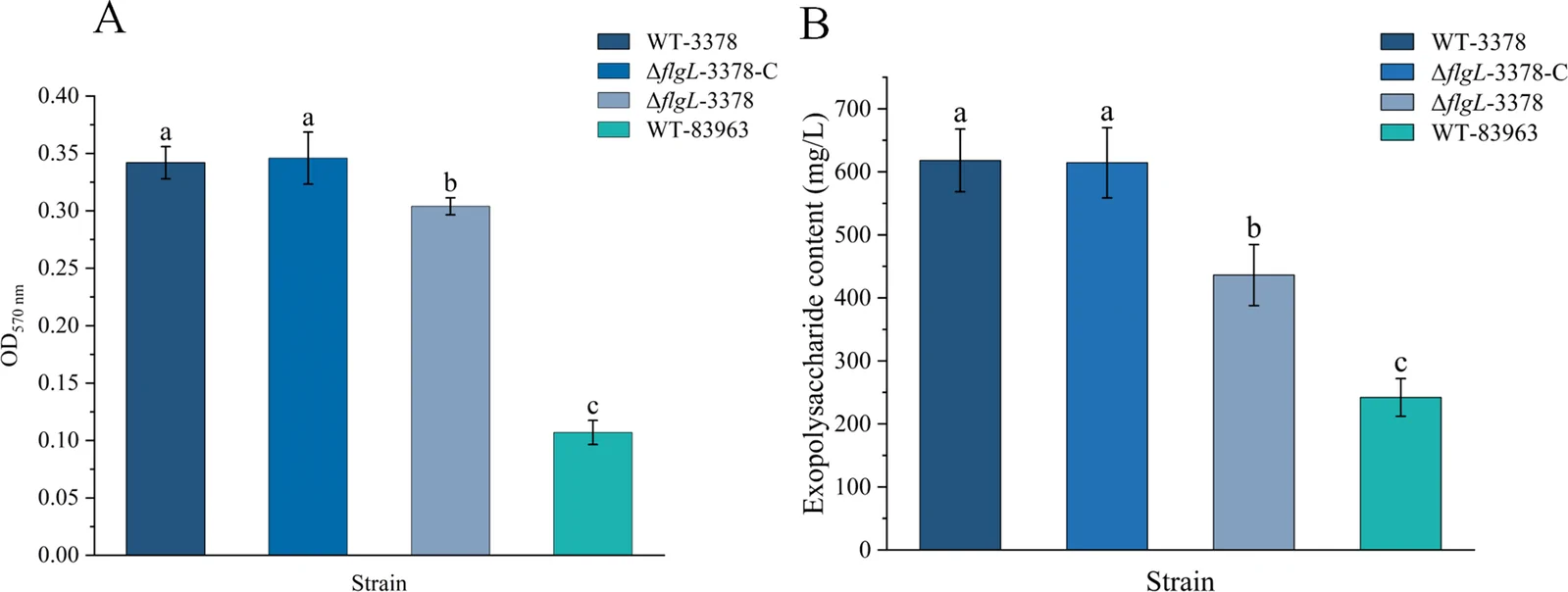
Biofilm formation (**A**) and extracellular polysaccharide production (**B**) of rhizobial strains *M. ciceri* USDA 3378 (WT-3378), *M. ciceri* 3378 deficient in *flgL* (Δ*flgL*-3378), Δ*flgL*-3378 complementation strain (Δ*flgL*-3378-C), and *M. muleiense* CCBAU 83963 (WT-83963). Data are presented as means (*n* = 3) ± standard deviations. Different letters indicate significant differences among treatments (*P* < 0.05), as determined by one-way analysis of variance.

Biofilm production by rhizobial strains decreased in the order WT-3378 (OD_570nm_ = 0.342 ± 0.01) > Δ*flgL*-3378 (0.304 ± 0.01) > WT-83963 (0.107 ± 0.01) (Fig. 4A). Extracellular polysaccharide production by rhizobial strains showed a similar order with WT-3378 (618.11 ± 49.71 mg/L) > Δ*flgL*-3378 (436.21 ± 48.41 mg/L) > WT-83963 (241.98 ± 29.94 mg/L) (Fig. 4B). Values for both biofilm production and extracellular polysaccharide production were similar for WT-3378 and Δ*flgL*-3378-C (Fig. 4A and B). These results indicate that the ability of *M. ciceri* to produce biofilm and extracellular polysaccharides is reduced by the deletion of *flgL* and that production of both is greater in WT-3378 than in WT-83963.

### Competitive nodulation ability of *M. ciceri* is diminished by deletion of *flgL*

**Fig 5:**
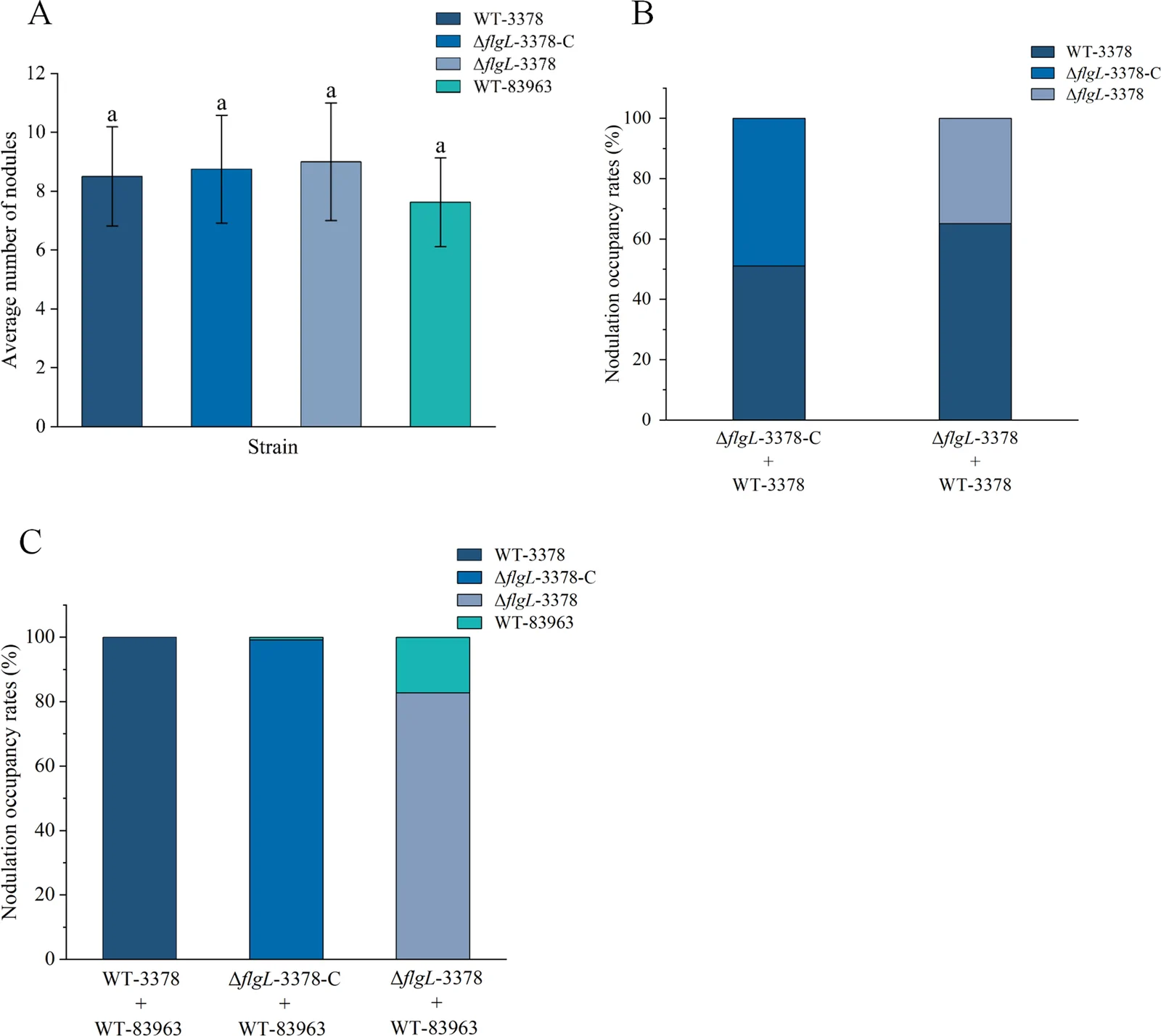
Nodule number of chickpea when inoculated individually (**A**) and percentage nodule occupancy when inoculated with selected pairings of rhizobial strains (**B and C**) *M. ciceri* USDA 3378 (WT-3378), *M. ciceri* 3378 deficient in *flgL* (Δ*flgL*-3378), Δ*flgL*-3378 complementation strain (Δ*flgL*-3378-C), and *M. muleiense* CCBAU 83963 (WT-83963). Data in panel A are presented as means (*n* = 8) ± standard deviations; the same letter for each data point indicates that there was no significant difference between treatments (*P* > 0.05), as determined by one-way analysis of variance.

Competitive nodulation on chickpea plants was assessed over 30 days using the strain combinations WT-3378 versus Δ*flgL*-3378 and WT-3378 or Δ*flgL*-3378 versus WT-83963. After 30 days, plants were harvested, and extracts from individual nodules served as DNA templates (Fig. S5A). Nodule occupancy by the different strains was subsequently determined by PCR (Fig. S5B) or BOX-PCR (Fig. S5C). The number of root nodules on chickpea plants was similar when inoculated with the different strains individually (Fig. 5A). Nodule occupancy by WT-3378 was 60.12% when co-inoculated with Δ*flgL*-3378 and 100% when co-inoculated with WT-83963 (Fig. 5B and C). However, nodule occupancy by Δ*flgL*-3378 was 82.80% when inoculated with WT-83963 (Fig. 5C). Thus, the competitive nodulation ability of *M. ciceri* was lower after *flgL* deletion. The complementation strain, Δ*flgL*-3378-C, restored the competitive nodulation ability of the mutant to a level close to that of WT-3378 (Fig. 5).

### Transcriptome profile of Δ*flgL*-3378 reveals altered flagellar and symbiosis-associated transcriptional responses

**Fig 6:**
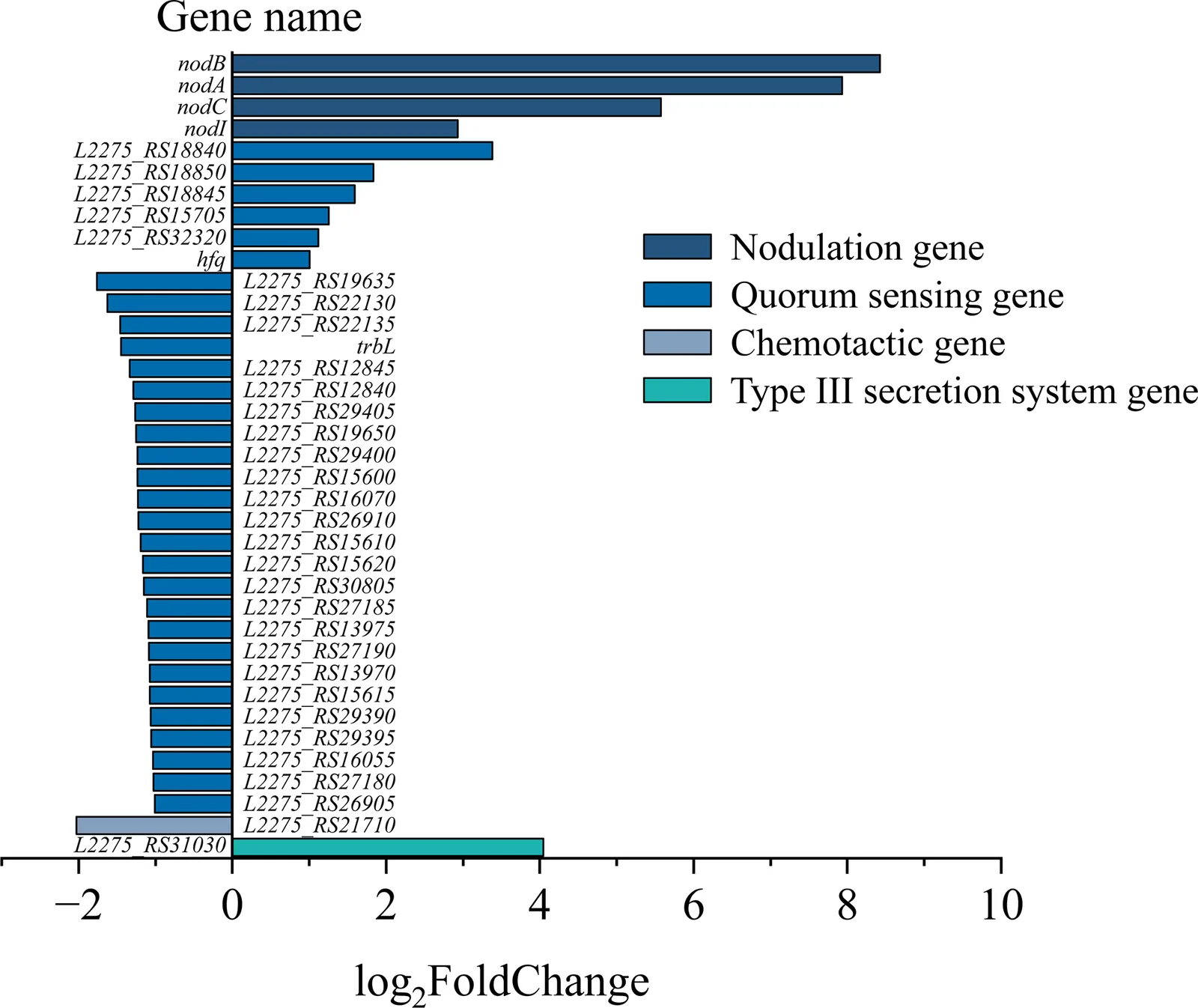
Differentially expressed genes related to nodulation, quorum sensing, and flagellar functions in Δ*flgL*-3378 under conditions that mimicked the symbiotic environment. Four flagellar system genes were significantly upregulated; among them, *flgK* (L2275_RS13320) and *flgE* (L2275_RS13325) were annotated as flagellar genes. Genes were considered differentially expressed with a *q*-value < 0.05 and |fold change| > 1.

To characterize the transcriptional response of the Δ*flgL*-3378 mutant under host-associated conditions, we analyzed its transcriptome under simulated symbiotic versus non-symbiotic conditions and compared the overall response pattern with that of WT-3378. As in WT-3378, several nodulation-related genes were significantly upregulated in Δ*flgL*-3378, indicating that deletion of *flgL* did not abolish perception of host-derived signals or induction of the nodulation program (Fig. 2 and 6). In addition, four flagellar system genes were significantly upregulated in Δ*flgL*-3378 under simulated symbiotic conditions. Among them, two annotated flagellar genes, *flgK* (L2275_RS13320) and *flgE* (L2275_RS13325), were both induced. Quorum-sensing-associated genes were also differentially expressed, whereas no chemotaxis-related genes met the threshold for differential expression under the conditions tested (Fig. 6). These results indicate that deletion of *flgL* altered, but did not eliminate, the symbiosis-associated transcriptional response.

## DISCUSSION

The objective of this study was to determine how the introduced *M. ciceri* USDA 3378 has a competitive advantage over the indigenous *M. muleiense* CCBAU 83963 in nodulating chickpea in soils in China. Initially, the genomes of the two strains were compared, focusing on gene clusters related to six factors reported to play an important role in the nodulation process: flagellum production, chemotaxis, quorum sensing, type III secretion systems, extracellular polysaccharide production, and nodulation gene systems. The only major difference between the two strains in this comparison was that the USDA 3378 genome contained 32 flagellum-related genes, divided into two clusters, one of which, the fla-1 gene cluster, was relatively well conserved, whereas the CCBAU 83963 genome contained only five flagellum-related genes scattered in different regions.

Evidence indicates that flagella can play an important role in nodulation. For example, deletion of the *flgE* gene in *Mesorhizobium tianshanense* resulted in loss of flagella and consequently complete loss of swimming ability, changes in nutrition-related clustering ability and biofilm formation, and decreased attachment to the root hairs of *Glycyrrhiza uralensis* (10). Also, flagellar genes *flaAB* and *flgK* positively influenced the competitiveness of colonization of *Medicago sativa* by *Sinorhizobium meliloti* (14). More recently, deletion of the *flgJ* in *Sinorhizobium fredii* and the associated loss of flagella did not affect its ability to colonize soybean (*Glycine max*) roots, but it did result in severe impairment of swimming and genistein-induced surface motility, and a reduction in symbiotic performance when not directly inoculated onto roots (15). In the current study, the transcriptome of USDA 3378 was sequenced under symbiotic and non-symbiotic conditions. Results showed that only one flagellum-related gene, *flgL*, was significantly upregulated in USDA 3378 under symbiotic conditions. Previous studies have shown that *flgL* is essential for the formation and assembly of polar flagella in the human and animal pathogen *Aeromonas hydrophila*, and *flgL*-deficient *A. hydrophila* had no flagella, and its swimming and adhesion abilities and virulence were decreased (16, 17). We hypothesized that flagellum-mediated motility could be the reason for the greater competitive nodulation ability of USDA 3378 compared to CCBAU 83963 for chickpea, and subsequent experiments using a targeted *flgL* deletion mutant (Δ*flgL*-3378) were designed to directly test this.

The *flgL* mutation did not affect the growth rate of the strain. This is consistent with previous studies showing that the deletion of *flgL* and *flgK* genes in *A. hydrophila* and the *flgJ* gene in *Sinorhizobium fredii* did not affect the growth of either bacterium (15, 17, 18). However, deletion of the *flgL* gene abolished the production of flagella, as confirmed by TEM, and significantly reduced motility. The wild-type USDA 3378 exhibited strong swimming and surface motility, whereas the Δ*flgL*-3378 mutant and the indigenous strain WT-83963 showed negligible expansion on soft agar plates, consistent with non-motile colony growth rather than active swimming. Also, our results showed that *flgL* expression is upregulated under symbiotic conditions, and motility is enhanced by root exudates. This mirrors findings in *Sinorhizobium fredii* HH103, where the flagellar hook gene *flgJ* is induced by host flavonoids (15), suggesting a conserved strategy in which rhizobia sense host signals to activate the flagellar apparatus. Furthermore, the complete loss of swimming motility and the marked reduction of surface motility in the Δ*flgL*-3378 mutant indicate that USDA 3378 relies on flagellum-dependent swarming motility. Rotating flagella provide the propulsive force for this energetically demanding process, while secreted surfactants (likely EPS or surface-active compounds) reduce surface tension (19, 20). This observation contrasts with *Sinorhizobium meliloti* 1021, which utilizes a flagellum-independent sliding mechanism driven solely by surfactants (21), but aligns partially with *S. fredii* HH103, which exhibits both swarming and sliding capabilities. Therefore, the surface translocation of USDA 3378 appears to be predominantly flagellum-dependent swarming (14, 22).

To facilitate swarming motility on surfaces, the cell also secretes mucoid layers, which function as surfactants or wetting agents. These agents reduce surface tension between adjacent bacteria, enabling effective collective movement (20). Biofilm and EPS production were both reduced in the Δ*flgL*-3378 mutant compared to WT-3378. However, biofilm and extracellular polysaccharide production by USDA 3378 and Δ*flgL*-3378 were higher than those by CCBAU 83963. It is not known how the *flgL* mutation/loss of flagella resulted in reduced biofilm and EPS production. Relations between flagella and biofilm and EPS production in rhizobia are not consistent across studies (10, 15). For example, in contrast to results here, the *flgJ* mutant of *Sinorhizobium fredii* showed increased ability to form biofilms, while its EPS production was similar to that of the wild-type strain (15).

Our study assessed the competitive nodulation ability of *M. ciceri* USDA 3378, its Δ*flgL*-3378 mutant, and CCBAU 83963 on chickpea plants over a 30-day period. Nodule occupancy by WT-3378 was 100% when co-inoculated with WT-83963 and 60.12% when co-inoculated with Δ*flgL*-3378. Δ*flgL*-3378 demonstrated a diminished competitive ability, occupying 82.8% of the nodules when co-inoculated with WT-83963. Furthermore, the Δ*flgL*-3378 complementation strain (Δ*flgL*-3378-C) exhibited a restored competitive nodulation ability, comparable to that of WT-3378. These observations provide strong evidence that the flagellar component gene *flgL* plays a role in the greater competitive nodulation ability of *M. ciceri* compared with *M. muleiense*. Previous studies indicate that rhizobial motility may serve as a key determinant for host root colonization and infection during early symbiotic stages (22). It is noteworthy that CCBAU 83963 naturally lacks flagella and flagellum-related genes, yet it retains the ability to nodulate chickpea efficiently in monoculture. This observation underscores that, while flagellar motility is not a prerequisite for nodulation, it is crucial for competitive success. Non-flagellated rhizobia, such as CCBAU 83963, likely rely on passive dispersal mechanisms—such as water percolation or physical displacement by root elongation—to contact root hairs. However, in a competitive environment where multiple strains coexist, the active motility confers a kinetic advantage, allowing the flagellated strain to reach and colonize infection sites prior to non-motile competitors. Furthermore, the observation that the non-motile Δ*flgL*-3378 mutant still outcompeted CCBAU 83963 (82.8% vs. 17.2%) suggests that, while flagella act as a powerful “colonization accelerator,” USDA 3378 possesses other intrinsic genomic advantages that contribute to its basal dominance over the indigenous strain. Research has shown that rhizobial surface polysaccharides (including EPS) can also act as signal molecules during the symbiotic interaction and/or as a protection against plant defenses (23, 24). The reduction in EPS and biofilm production may also be a contributing factor to the impaired competitive nodulation ability of the Δ*flgL*-3378 mutant.

Transcriptome analysis further suggests that the consequences of *flgL* deletion extend beyond the physical loss of flagella. A notable finding was that, under symbiotic conditions, four flagellar system genes were significantly upregulated in Δ*flgL*-3378, including the annotated genes *flgK* (L2275_RS13320) and *flgE* (L2275_RS13325), whereas these genes were not significantly altered in WT-3378. Given that FlgL functions at the hook-filament junction, this pattern is more consistent with compensatory or feedback responses associated with incomplete flagellar assembly than with direct transcriptional control by FlgL itself. At the same time, several nodulation-related genes were differentially expressed in both WT-3378 and Δ*flgL*-3378, indicating that deletion of *flgL* did not abolish the symbiosis-responsive nodulation transcriptional program, although it may have altered its expression pattern. In addition, quorum-sensing-associated genes were differentially expressed in the mutant, whereas no chemotaxis-related genes met the threshold for differential expression under the conditions tested. These results suggest that the reduced competitive nodulation ability of Δ*flgL*-3378 is unlikely to be explained by chemotaxis-related transcription alone. Rather, it is more plausibly attributable to the combined effects of impaired flagellar assembly, reduced motility, altered biofilm and extracellular polysaccharide production, and reshaped symbiosis-associated transcriptional responses.

In summary, our work provides strong evidence that *flgL* contributes to the superior competitive nodulation ability of *M. ciceri* USDA 3378 over *M. muleiense* CCBAU 83963 on chickpea. Loss of *flgL* abolished flagellum formation, reduced motility, biofilm formation, and extracellular polysaccharide production and was accompanied by altered symbiosis-associated transcriptional responses. Future work should identify the additional motility-independent traits that contribute to the basal competitive advantage of USDA 3378.

## MATERIALS AND METHODS

### Bacterial strains, plasmids and growth conditions

Bacterial strains and plasmids used in this study are listed in Table 1. The *Escherichia coli* strains of DH5α and S17-1 were grown at 37°C in liquid Luria-Bertani (LB) medium (25) or LB with 1.8% agar (depending on the experiment), containing kanamycin and gentamicin at 50 and 30 μg/mL, respectively. *M. ciceri* USDA 3378 and *M. muleiense* CCBAU 83963 were grown at 28°C in liquid Tryptone-Yeast (TY) medium (26) or Yeast-Mannitol Agar (M-YMA) medium (27), or on TY or M-YMA medium supplemented with 1.8% agar. Plasmids were introduced into *E. coli* by heat shock at 42°C and into USDA 3378 through biparental mating (28, 29).

**TABLE 1:** Bacterial strains and plasmids used in this study

Strain or plasmid	Characteristics	Reference or source
Strains		
*M. ciceri* USDA 3378	Isolated from chickpea in Japan; Kan^r^	Lab stock
*M. muleiense* CCBAU 83963	Isolated from chickpea in Xinjiang, China; Chl^r^	China Agricultural University
Δ*flgL*-3378	*flgL* mutant strain based on USDA 3378; Kan^r^, Gm^r^	This study
Δ*flgL*-3378-C	USDA 3378 flgL complemented strain; Kan^r^, Gm^r^	This study
*E. coli* DH5α	lac ZΔM15, recA gyrA	TaKaRa
*E. coli* S17-1	recA pro (RP4-2Tet::Mu Kan::Tn7)	Huazhong Agricultural University
Plasmids		
pCM351	Cre-loxP recombinant system plasmid vector; Gm^r^	Huazhong Agricultural University
pCM351::flgLup-down	pCM351 with upstream and downstream homologous arms of *flgL*; Gm^r^	This study
pCM157	Cre recombinase bacterial expression; Tet^r^	Huazhong Agricultural University
pBBR1MCS-5	Broad-spectrum host plasmid; Gm^r^	Huazhong Agricultural University
pBBR1MCS-5-flgL	pBBR1MCS-5 with complementary sequence of *flgL*; Gm^r^	

### Comparison of gene clusters in USDA 3378 and CCBAU 83963 associated with competitive nodulation

USDA 3378 and CCBAU 83963 were inoculated into M-YMA liquid medium and cultured on a shaker (180 rpm) at 28°C to the logarithmic growth phase. The cell biomass was harvested after 10 min of centrifugation at 12,000 × *g* at 4°C. Genomic DNA of USDA 3378 and CCBAU 83963 was extracted using a bacterial DNA extraction kit (Majorbio, Shanghai, China). Purified genomic DNA was quantified using a NanoDrop 2000 to detect the absorbance of DNA at 260 nm, and the purity was evaluated by OD_260nm_/OD_280nm_ ratio. High-quality DNA was used in further research. The genomic DNA was sheared into 400 bp fragments (Covaris M220 Focused Acoustic Shear), and a NEXTFLEX Rapid DNA-Seq Kit was used to prepare a DNA library for paired-end Illumina sequencing (2 × 150 bp) on a NovaSeq 6000 platform (Illumina Inc., San Diego, CA, USA). The data generated were used for bioinformatics analysis. All analyses were performed using the online Majorbio Cloud Platform (Shanghai Majorbio Bio-pharm Technology Co., Ltd.; https://v.majorbio.com/).

The distribution and number of functional genes on the genomes of *M. ciceri* USDA 3378 and *M. muleiense* CCBAU 83963 were compared, and gene clusters associated with competitive nodulation ability were visualized using the Genelibs website (https://www.genelibs.com/gb/pages/login.jsf).

### Transcriptome sequencing under symbiotic and non-symbiotic conditions

Commercially obtained Kabuli chickpea (*Cicer arietinum* L.) seeds grown in Xinjiang, China, were placed in an ethanol-washed conical bottle containing 2.5% sodium hypochlorite solution for 4 min, washed with sterile water seven times, and germinated in water in the dark at 28°C for 4 days. Chickpea sprouts showing uniform growth were added to a 50 mL bacterial suspension of USDA 3378 (OD_600nm_ = 1.0) in 100 mL bottles. Twelve chickpea sprouts were added to each bottle of bacteria and cultured at 28°C and 50 rpm for 2 h to simulate symbiotic conditions (8, 30). USDA 3378 was cultured similarly without chickpea sprouts to simulate non-symbiotic conditions. The bacteria were collected by centrifugation at 12,000 rpm (14,009 × *g*) and 4°C for 5 min. For transcriptome analysis of wild-type USDA 3378, three biological replicates were included for the root exudate treatment, and two biological replicates were available for the non-symbiotic control condition. For transcriptome analysis of Δ*flgL*-3378, three biological replicates were included for each condition. Bacteria were stored at −80°C. The Δ*flgL*-3378 mutant was also subjected to transcriptome sequencing under symbiotic and non-symbiotic conditions using the same method.

Total RNA was extracted using a Total RNA Extractor (Trizol) Kit (B511311, Sangon, China) and treated with RNase-free DNase I to remove genomic DNA contamination. RNA integrity was evaluated on a 1.0% (wt/vol) agarose gel. Thereafter, the quality and quantity of RNA were assessed using a NanoPhotometer spectrophotometer (IMPLEN, CA, USA) and a Qubit 2.0 Fluorometer (Invitrogen). High-quality RNA samples were subsequently submitted to Sangon Biotech (Shanghai) Co., Ltd. for library preparation and sequencing. A total of 1 μg RNA per sample was used as input material for RNA sample preparations. Sequencing libraries were generated using a VAHTS mRNA-seq V2 Library Prep Kit for Illumina, and index codes were added to attribute sequences to each sample. Library quality was assessed using an Agilent Bioanalyzer 2100 system, and libraries were then quantified and pooled. Paired-end sequencing of libraries was performed using NovaSeq sequencers (Illumina, San Diego, CA). Clean reads were mapped to the reference genome using HISAT2 (version 2.0) with default parameters. RSeQC (version 2.6.1) (available from http://rseqc.sourceforge.net/) was used to statistically analyze the alignment results. DESeq2 (version 1.12.4) (available via Bioconductor: https://bioconductor.org/packages/DESeq2) was used to determine differentially expressed genes (DEGs) between the two samples. Genes were considered differentially expressed with a *q*-value ≤0.05 and |fold change| ≥ 1. DEGs were mapped to Gene Ontology (GO) terms (biological functions) in the database, the number of genes associated with each term was calculated, and a hypergeometric test was performed to identify significantly enriched GO terms in the gene list relative to the background of the reference gene list. KEGG pathway analysis was used to identify metabolic pathways or signal transduction pathways significantly enriched in DEGs compared with a reference gene background using the hypergeometric test. Genes of interest were identified through their functional description, and annotation was carried out on the DEGs.

### Construction of *flgL* gene mutant strain and its genetically complemented strain

The upstream and downstream homologous arms of *flgL* were amplified using *flgL*-up-F/R (*Kpn*I, *Nde*I) and *flgL*-down-F/R (*Apa*I, *Sac*I) primers with the wild-type USDA 3378 (WT-3378) genome as a template. The upstream and downstream homologous arms and plasmid pCM351 were then digested, purified, and connected step-by-step using a Hieff Clone Universal One Step Cloning Kit to obtain the recombinant plasmid pCM351::*flgL*up-down, which was sequenced. A plasmid containing the correct sequence was transformed into *E. coli* S17-1, and the resulting strain was conjugated with WT-3378. Double-exchange mutants were identified by screening on TY agar plates supplemented with kanamycin (30 μg/mL) and gentamicin (30 μg/mL). Primers 13315-F and 13315-R were used for PCR verification of mutants.

Δ*flgL*-3378 was conjugated with *E. coli* S17-1 containing plasmid pCM157, and Δ*flgL*-3378 strains that had lost the gentamicin resistance gene were identified by screening on TY agar plates with kanamycin (30 µg/mL) and tetracycline (20 µg/mL) added. Primers 13315 F/R were used to verify colonies of Δ*flgL*-3378 without the gentamicin resistance gene. The complete *flgL* gene sequence (including the 600 bp sequence upstream of the start codon) was amplified with GH-F/R (*Apa*I, *Sac*I) primers using the WT-3378 genome as the template. The complementary *flgL* sequence was purified and connected to pBBR1MCS-5 linearized by double endonuclease digestion using a Hieff Clone Universal One Step Cloning Kit to obtain the recombinant plasmid pBBR1MCS-5-*flgL*. Colony PCR was performed with universal primers M13F/R to verify whether the complementary sequence was successfully linked and sequenced. A plasmid containing the correct sequence was transformed into *E. coli* S17-1, and the resulting strain was conjugated with Δ*flgL*-3378; complementation strains of Δ*flgL*-3378 were screened on TY agar plates containing kanamycin (30 µg/mL) and gentamicin (30 µg/mL). Finally, the complementation strains were verified by PCR and sequenced using primers M13F/R. All primers used in this study are listed in Table 2.

**TABLE 2:** Primers used for construction of flgL mutant and complementary strains

Primer	Sequence (5′–3′)	Fragment size (bp)	Target gene
*flgL*-up-F	CTGAATTCAGCTGTACAATTGGTACCTGTTCACCTGGTCGGGAG	516	*flgL* upstream homology arm
*flgL*-up-R	ATACGAAGTTATGCGGCCGCCATATGGGAATTATCCCACCGCGTTG		
*flgL*-down-F	ATCCAGCTTATCGATACCGCGGGCCCTTAACGGAACGGTAGAGCCC	503	*flgL* downstream homology arm
*flgL*-down-R	GGTCGGCTGGATCCTCTAGTGAGCTCATCACGTGGCTTTCGAGCAG		
13315-F	ACATCGACAACGTGCCGATC	2,298 (Δ*flgL*-3378)/2,426 (WT-3378)	Whole region of *flgL* for identification
13315-R	TCGATCTGTTTCAGCGCGCT		
GH-F	ACTCACTATAGGGCGAATTGGAGCTCTCGACCATGCAGAGCCAG	1,657	Complementary sequence of *flgL*
GH-R	GGGAACAAAAGCTGGGTACCGGGCCCGCGTTGTCAGGTCAGGTAAT		
M13F	CGCCAGGGTTTTCCCAGTCACGAC		
M13R	AGCGGATAACAATTTCACACAGG		

### Construction of growth curves

Growth curves for Δ*flgL*-3378, WT-3378, and wild-type CCBAU 83963 (WT-83963) strains were generated to determine if the Δ*flgL* mutation affects the growth of *M. ciceri*. Cultures of the three strains in M-YMA liquid medium were adjusted to the same bacterial density before being inoculated into fresh M-YMA liquid medium at a rate of 1% and cultured at 28°C with shaking at 180 rpm. Triplicate samples were collected at intervals of 12 h to measure bacterial concentration and to construct a growth curve.

### Motility test

The motility of Δ*flgL*-3378, WT-3378, and WT-83963 was determined in semisolid culture medium (31). All three strains were inoculated into 5 mL of TY broth and cultured at 28°C with shaking at 180 rpm until an OD_600nm_ of 1.0 was reached. The same volume (3 μL) of each strain was aspirated and inoculated into 0.3% agar BM medium. The plate was sealed with Parafilm, incubated upright at 28°C for 24 h, then inverted and further incubated at 28°C for an additional 4 days. Finally, the swimming motility distance of the strains was measured. Each test was performed in triplicate.

Additionally, a surface motility assay was performed. The three test bacterial strains were cultured in TY broth to an OD_600nm_ of 1.0, using the same method as described for the swimming motility assay. A 1 mL bacterial culture was transferred to a sterile 1.5 mL microcentrifuge tube and centrifuged at 12,000 × *g* for 3 min. The pellet was washed twice with 1 mL of liquid MM medium, the supernatant was discarded, and the pellet was gently resuspended in 100 µL of liquid MM medium. A 2 μL aliquot of the bacterial suspension was spotted onto the center of a 0.4% agarose-MM medium plate. After drying for 10 min at room temperature, the plate was sealed with Parafilm and incubated inverted at 28°C for 5 days. Following 120 h of incubation at 28°C, plates were stored at 4°C for 2 days prior to motility distance measurement. Each test was performed in triplicate.

To investigate the effect of host signals on motility, unconcentrated chickpea root exudates were collected and added to BM and MM semisolid media at a final concentration of 4% (vol/vol). Swimming and surface motility assays were then performed on these plates following the same protocols described above.

### Transmission electron microscopy

Cells of the Δ*flgL*-3378, WT-3378, and WT-83963 strains were observed using a transmission electron microscope (Tecnai 12, Philips Company, Holland) as described previously (32). Briefly, a drop of bacterial solution was placed on a slide and covered with a copper mesh for 2 min. The copper mesh was then removed and placed on a tray at room temperature for 3 min before any excess bacterial solution was removed using absorbent paper. A drop of 5% phosphotungstic acid negative stain was then placed on the copper mesh. Excess dye was pipetted off after 1 min, and the copper mesh was washed with deionized water and dried overnight. Samples were observed using a transmission electron microscope.

### Measurement of biofilm production

Biofilm production by Δ*flgL*-3378, WT-3378, and WT-83963 was determined as described previously (8). The three strains were inoculated into 5 mL of M-YMA liquid medium, cultured to log phase, and adjusted to the same density (OD_600nm_ = 1.0) as above. A 0.15 mL sample of each culture was inoculated into 1.5 mL of M-YMA liquid culture and maintained at 28°C for 7 days. The bacterial liquid in the tube was discarded, and the tube was washed three times with deionized water. Two milliliters of 0.1% (wt/vol) crystal violet dye solution was added to the test tube and incubated for 30 min to stain the biofilm; the dye solution was then discarded. The tube was washed three times with deionized water and then air-dried upside down to allow biofilm formation on the inner wall of the test tube. The biofilm was solubilized in 3 mL of 30% (vol/vol) acetic acid solution, and the absorbance was recorded at 570 nm. The test was performed in triplicate.

#### Determination of extracellular polysaccharide production

The extracellular polysaccharides (EPS) produced by Δ*flgL*-3378, WT-3378, and WT-83963 were quantified using the phenol-sulfuric acid method (33). The three strains were cultured in M-YMA liquid medium and adjusted to the same density (OD_600nm_ = 1.0) as described above. The cultures were centrifuged at 4°C and 4,000 rpm (1,887 × *g*) for 15 min, and the supernatant was collected. Ethanol (96%, vol/vol) was added to the supernatant at a ratio of 4:1 (vol/vol), and the solution was mixed and left to stand at 4°C for 30 min, during which time white flocs (extracellular polysaccharide) formed in the tube. The supernatant was discarded after centrifugation at 4°C and 10,000 rpm (11,791 × *g*) for 15 min, and the precipitate was air-dried (~ 8 h). The dried extracellular polysaccharide was dissolved in 5 mL deionized water. Finally, 50 µL of the sample solution was combined with 950 µL of DDH₂O in a test tube. Subsequently, 0.5 mL of 6% (wt/vol) aqueous phenol solution (freshly prepared) was added, followed immediately by the rapid addition of 5 mL of concentrated sulfuric acid. The reaction mixture was incubated at room temperature for 20 min, and the OD_490nm_ value of each sample was determined. The content of extracellular polysaccharides produced by the three strains was calculated by reference to a glucose standard curve. Measurements were performed in triplicate.

### Determination of competitive nodulation ability

The competitive nodulation ability of Δ*flgL*-3378 versus WT-3378 and of Δ*flgL*-3378 or WT-3378 against WT-83963 was assessed in plant tray assays. Cultures of the three strains in M-YMA liquid medium were adjusted to the same density. Each two-strain inoculation treatment was performed on Kabuli chickpea sprouts (seed-surface sterilized at sowing) grown in sterilized plastic trays (15 cm × 10 cm) filled with sterilized vermiculite (34): (i) 1 mL Δ*flgL*-3378 and 1 mL WT-3378 in mixed solution; (ii) 1 mL Δ*flgL*-3378 and 1 mL WT-83963 in mixed solution; and (iii) 1 mL WT-3378 and 1 mL WT-83963 in mixed solution. The three strains were also tested individually. Ten replicate plants under each treatment were grown in a glasshouse at 25/20°C day/night temperature with a photoperiod of 16 h. After 30 days, the nodules from each plant were harvested, disinfected (soaked in 2.5% sodium hypochlorite solution for 3 min), and crushed. In the competitive nodulation assay between Δ*flgL*-3378 and WT-3378, nodule extracts were used as DNA templates for PCR amplification with primers 13315F/13315R to determine the occupying strain for each individual nodule. For competitive nodulation assays between either WT-3378 or Δ*flgL*-3378 against WT-83963, extracts from each nodule were used as DNA template for BOX-PCR amplification using the unidirectional primer BOX-A1R to determine which strain formed each nodule.

### Verification of phenotypic recovery of Δ*flgL*-3378 complementation strains

The phenotypic recovery of the Δ*flgL*-3378 complementation strain (Δ*flgL*-3378-C) was verified by assessing motility, growth curve, biofilm formation ability, exopolysaccharide production, and competitive nodulation ability as above.

### Statistical analysis

Analysis of variance was performed using SPSS v.21.0 software. Results are presented as mean ± standard deviation. Each experiment was repeated at least three times. Differences were considered significant when *P* < 0.05.

## Data Availability

Whole-genome sequencing data of *M. ciceri* USDA 3378 and *M. muleiense* CCBAU 83963 were uploaded to the NCBI database under accession numbers NZ_JAKHFU010000000.1 and NZ_FNEE01000000.1, respectively. The raw RNA-seq reads generated in this study have been deposited in the NCBI Sequence Read Archive (SRA). The wild-type USDA 3378 data sets are available under accession numbers SRR38125703, SRR38125702, SRR38125701, SRR38125700, and SRR38125699. The Δ*flgL*-3378 data sets are available under accession numbers SRR37914422, SRR37914421, SRR37914420, SRR37914419, SRR37914418, and SRR37914417. Additional data supporting the findings of this study are available within the article and its supplemental material.

## SUPPLEMENTAL MATERIAL

Supplemental material aem.00931-26-s0001.docx Table S1; Fig. S1 to S5.

ASM does not own the copyrights to Supplemental Material that may be linked to, or accessed through, an article. The authors have granted ASM a non-exclusive, world-wide license to publish the Supplemental Material files. Please contact the corresponding author directly for reuse.
