# Supplementary material for: Deletion of flgL in Mesorhizobium ciceri USDA 3378 weakened competitive nodulation ability by reducing flagellum formation, biofilm formation, and extracellular polysaccharide secretion
Source: Appl Environ Microbiol. 2026 Jul 10;92(8):e00931-26. doi: 10.1128/aem.00931-26 (PMC13488323; doi:10.1128/aem.00931-26)
Supplement: Supplementary file 1 [file aem.00931-26-s0001.docx]

**Supplementary Materials：**

| Strain | Gene entry number | Gene | Initiation | Terminate | Direction | Base number |
| --- | --- | --- | --- | --- | --- | --- |
| USDA 3378 | JAKHFU010000001 | *CheBR* | 306571 | 309615 | + | 3045 |
|  | JAKHFU010000004 | *CheY* | 228254 | 228658 | + | 405 |
| CCBAU 83963 | FNEE01000001 | *CheBR* | 359870 | 362980 | + | 3111 |
|  | FNEE01000008 | *CheY* | 69068 | 69436 | + | 369 |

**Table S1** Chemotactic genes of two chickpea rhizobi

**Fig. S1** Comparison of nodulation related gene clusters of two chickpea rhizobia USDA 3378 and CCBAU 83963. **A**. Quorum sensing gene clusters. **B**. Exopolysaccharide gene clusters. **C**. Type III secretion gene clusters. **D**. Nodulation gene clusters.

**Fig. S2** Construction of Δ*flgL*-3378 strain. **A** Amplification of upstream homologous arm and downstream homologous arm. M: DL2000 marker. 1: upstream homologous arm. 2: downstream homologous arms. **B,** **C** The recombinant products were transformed into *E*. coli DH5a. M: DL5000 marker. M: DL5000 marker. 3,4: PCR results of upstream homologous arm were verified. 5,6: PCR results of downstream homologous arms were verified. **D** Mutant screening results M: DL5000 marker. 7: Wild strain as control. 8: Mutant strain after homologous recombination double exchange.

**Fig. S3** Construction of complementary strain of Δ*flgL*-3378 **A** Screening results for ∆*flgL*-3378 without gentamicin resistance. M:DL5000 marker 1: Δ*flgL*-3378 2: Elimination of Δ*flgL*-3378 of gentamicin resistance gene **B** Verification results of successful ligation of recombinant plasmids. M: DL2000 marker 3-5: Complementary sequences of Δ*flgL*-3378 **C** Verification results of Recombinant plasmid transferred into Δ*flgL*-3378 M: DL2000 marker 6-8: Complementary sequences of Δ*flgL*-3378.

**Fig. S4** Host signals affect motility tests. A: Swimming motility assays of WT-3378 and Δ*flgL*-3378. Panel B: Surface motility assays of WT-3378 and Δ*flgL*-3378. In both A and B, the right-hand side corresponds to experiments conducted in medium supplemented with root exudates.

A

C

B

**Fig. S5** Nodulation of chickpea plants and PCR amplifications or fingerprints to identify different nodules. **A** Nodulation of some chickpea plants inoculated with different strains. Bars =15 cm. **B** Results of PCR fingerprint of partial competitive nodulation. 1: PCR amplification bands of Δ*flgL*-3378 2: PCR amplification bands of WT-3378 M: DL 5000 marker. **C** Results of BOX-PCR fingerprint of partial competitive nodulation.3: BOX-PCR fingerprint of USDA 3378; 4: BOX-PCR fingerprint of CCBAU 83963 M: DL 5000 marker
